# Industrial Dentistry’s Contribution to Oral Hygiene

**Published:** 1923-10

**Authors:** R. I. Humphrey

**Affiliations:** Chicago, Illinios


### INDUSTRIAL DENTISTRY'S CONTRIBUTION TO ORAL HYGIENE *

BY R. I. HUMPHREY, D. D. S., CHICAGO, ILLINIOS

In considering the subject of industrial dentistry and its value to general health, one can not help but feel a sense of disappointment at the extremely bad condition found in the mouths of people who are working in our densely populated centers. We who have been engaged in industrial work during the past few years are probably the only ones in our profession who fully realize the extremely insanitary condition which actually exists. Oral hygiene among this element of our population has been so neglected that one sometimes wonders that the condition is not even worse than we find it to be. Many of these people are uneducated and especially so in the field of oral hygiene. The importance of such a common operation as brushing their teeth appears never to have entered their minds. Neither have they gone to dentists for prophylactic or constructive dental care, except perhaps as the result of an emergency such as a pulpitis or an acute abscess, and the only treatment many of them then received was the removal of the offending tooth.

Considering the cause of this most unhealthy condition, we should not overlook the fact that more than fifty per cent. of the employes whose mouth condition we have recorded are foreign born, and that more than ninety per cent. of this number come from rural districts in their own countries. From these findings it would seem that the people of rural districts are considerably less informed regarding mouth hygiene, especially among the foreign born.

Industrial oral hygiene seems to be the missing link between the care of the mouths of school children and of the better educated, more or less well-to-do people who receive periodic prophylaxis. We know that it is a comparatively simple matter to serve patients who have been taught the importance of oral hygiene from childhood, and who have the means to secure first-class service. However, this is not the case in the industrial field. We must first in some way bring to the minds of our patients the importance of keeping the mouth clean, and it is this particular phase of the work which seems to hold the greatest promise in industrial dental service. It is not a simple task to persuade all those adults with whom we come in contact to follow out a definite prophylactic program. However, our results have been encouraging enough to warrant the continuation of this service, and if possible, to add to it the support of more dentists in private practice.

The field of industrial dentistry thus far has not received adequate support from physicians and dentists engaged in private practice. It seems that there are still a few private practitioners who hold the view that the effect of industrial work is to rob them of part of their livelihood. However, when we stop to consider this phase of the situation, we know that only a limited amount of constructive work could possibly be accomplished by men in industry, even if the number of dentists engaged in this type of work were many times greater than at present. Furthermore, the value which ultimately accrues to the private practitioner through the education of employes along this line is very considerable. When the head of the family has been convinced that the care of the mouth is highly important, he will naturally insist that other members of his family are properly cared for.

Physicians in industry are constantly coming in contact with chronic cases where oral hygiene has a direct bearing on the prognosis in the case. The close cooperation of physician and dentist in cases of this kind is absolutely necessary if satisfactory results are to be obtained. This, too, is one of the big fields in industrial dentistry, and one which is producing very remarkable results.

The following cases are typical of many which we have treated during the past six years:

Case No. 1. Male, age 35. Family and personal history negative. Patient complained of pain in muscles of neck and shoulders and had not slept well for two weeks. Physician could not find anything which would account for the trouble. Oral examination revealed four deep pyorrhea pockets. The affected teeth were extracted followed by the immediate disappearance of muscle pain.

Case No. 2. Female, age 31. Family and personal history negative. Patient complained of pain in stomach two years previous. Physician sent patient to hospital for observation followed by a diagnosis of probable gastric ulcer. Diet was recommended and after a period of six weeks patient returned to work feeling greatly improved. After about six months patient had same trouble and was sent to general hospital a second time for diagnosis. This time all the findings were negative except six teeth which were abscessed. The teeth were extracted followed by the patient's return to work and in six months the patient gained twenty pounds. Three years have elapsed since treatment, during which time there has been no symptoms of the old trouble.

Case No. 3. Male, age 37. Family and personal history negative. Patient complained of excruciating pain in left thigh of two weeks' duration. Physician's diagnosis was sciatica. Examination of mouth revealed extremely septic condition, including pyorrhea, abscesses and caries. All teeth were extracted followed by a very rapid improvement. There has been no recurrent trouble during the past four years.

A great deal has been said regarding focal infection and its relation to general health, and in some cases perhaps there has been a degree of exaggeration. However, after handling severeal hundred of these patients, it is really astonishing to note the vast improvement in their general health following the elimination of mouth infection.

It is a somewhat debatable question just how far we should go in the way of restorative work in the industrial field, because conditions in different localities and in different industries are not the same. However, it seems to me that in addition to the educational feature, which must always predominate, we should include such service as the immediate relief of pain, cleansing of teeth, extractions, abscess treatment, X-ray, and at least a limited amount of filling and plate work.

The two most vital points which we should always consider, are the prevention of pulp exposure and the prevention and elimination of periodontal infection. After all, industrial oral hygiene is no different from any other field of dentistry, in that the object sought is the rendering of the maximum service, and it has been the aim from the very outset of this most worthy movement to give the greatest service possible that is compatible with existing conditions.

It has been a source of considerable satisfaction to know that employers have co-operated very thoroughly in industrial dentistry. We hope that our dental organizations will take a more active and constructive attitude in helping us to render a better and more complete service to a large body of our population who are badly in need of dental service, and who were almost entirely neglected until industrial dentistry was inaugerated a few years ago.—*American Dental Journal.*

